# A case of carcinoma ex pleomorphic adenoma arising from multinodular pleomorphic adenoma of the buccal region

**DOI:** 10.1002/ccr3.2417

**Published:** 2019-09-10

**Authors:** Koji Yamamoto, Yuichiro Sato, Yuudai Kondo, Hiroyuki Tanaka, Yutaka Akiyama, Yoshihiro Yamashita, Hiroaki Kataoka

**Affiliations:** ^1^ Section of Oncopathology and Regenerative Biology Department of Pathology Faculty of Medicine University of Miyazaki Miyazaki Japan; ^2^ Department of Diagnostic Pathology Miyazaki University Hospital University of Miyazaki Miyazaki Japan; ^3^ Department of Oral and Maxillofacial Surgery Faculty of Medicine University of Miyazaki Miyazaki Japan

**Keywords:** carcinoma ex pleomorphic adenoma, fine‐needle aspiration, minor salivary gland tumor, multinodular lesion, salivary duct carcinoma

## Abstract

In multinodular lesions or tumors with mixed benign and malignant components, malignant elements may be undetectable using fine‐needle aspiration biopsy/cytology in preoperative pathological diagnosis of some cases, because of sampling error.

## 1 INTRODUCTION

Carcinoma ex pleomorphic adenoma (PA) (Ca‐ex‐PA) is a rare epithelial malignancy arising from primary or recurrent PA. It comprises approximately 3.6% of all salivary gland tumors and 12% of all salivary malignancies.^1^, ^2^, ^3^ The common sites of origin are the major salivary glands, most commonly the parotid gland, followed by the submandibular gland. In the minor salivary glands, Ca‐ex‐PA frequently occurs in the palate gland, whereas in the buccal mucosa, it is rare. For the preoperative diagnosis of Ca‐ex‐PA, incision biopsy, fine‐needle aspiration (FNA) biopsy (FNAB), and FNA cytology (FNAC) are widely performed. FNAB and FNAC are relatively easy, inexpensive, and quick to perform and are well accepted by patients,^4^, ^5^ but their sensitivities are low owing to sampling error because the samples are very small. We report a case wherein a malignant component was identified through pathological examination after surgery, although the patient was diagnosed with PA based on preoperative FNAB.

## 2 CASE HISTORY AND EXAMINATION

**Figure 1:**
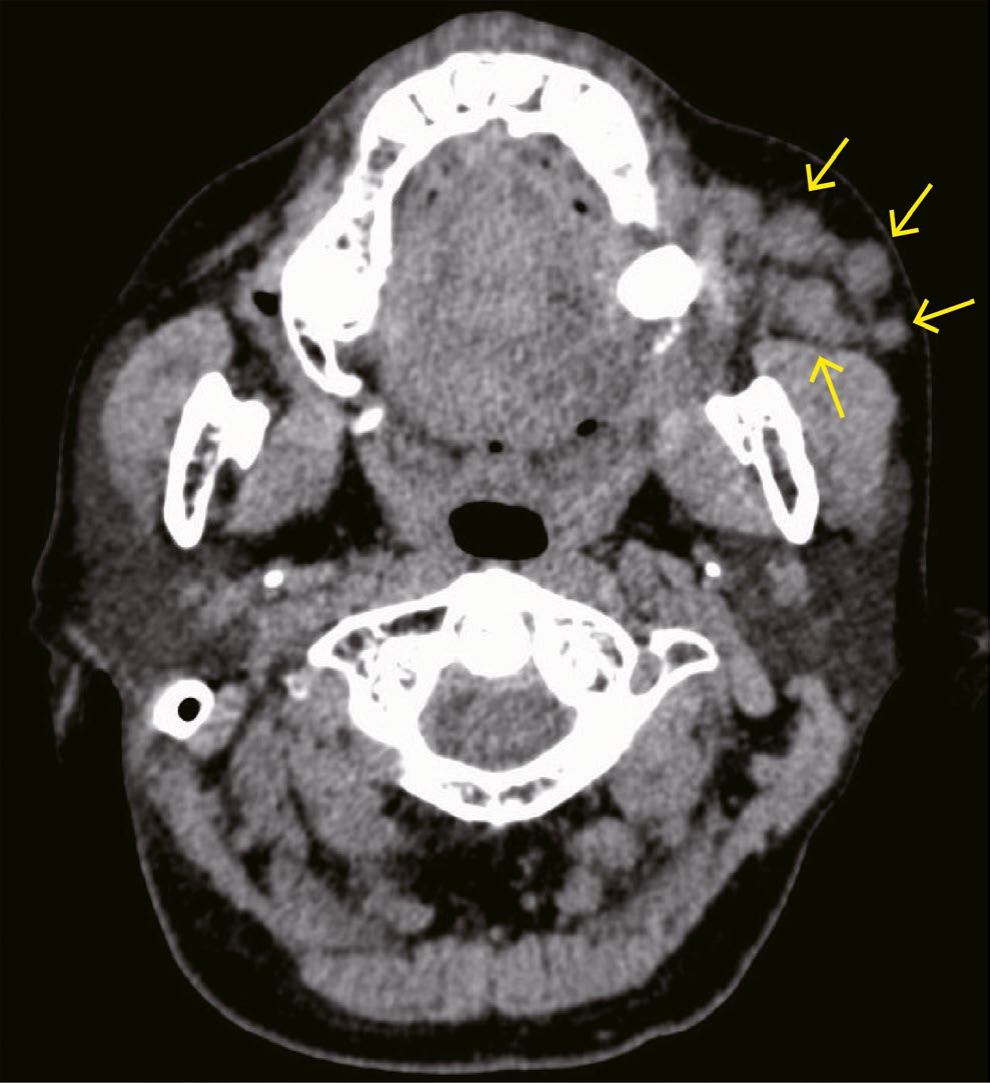
Preoperative nonenhanced CT image. Under the left cheek, some irregular tumor masses were observed (arrows). CT, computed tomography

**Figure 2:**
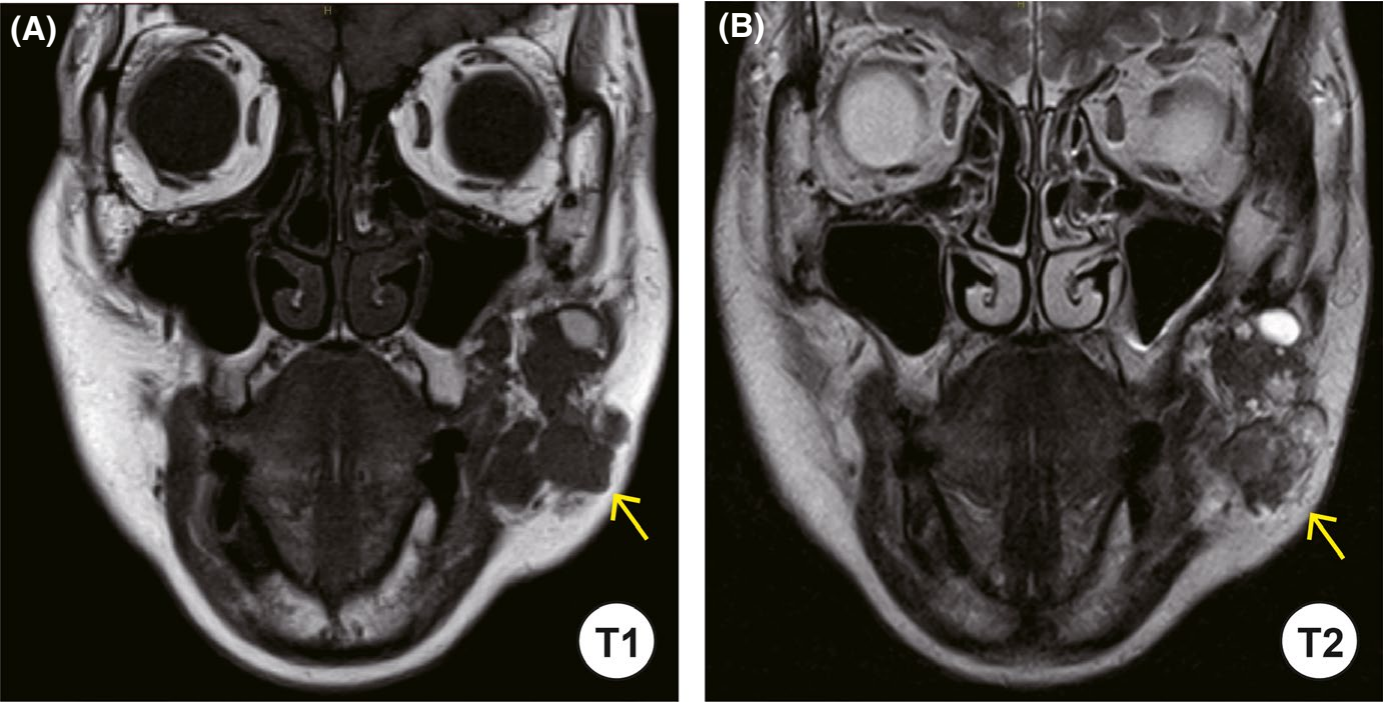
T1‐ and T2‐weighted MRI of the tumor masses. MRI showed low‐intensity nodules on T1‐weighted (A) and heterogeneous nodules with mixed high‐ and low‐intensity signals on T2‐weighted (B) images. MRI, magnetic resonance imaging

**Figure 3:**
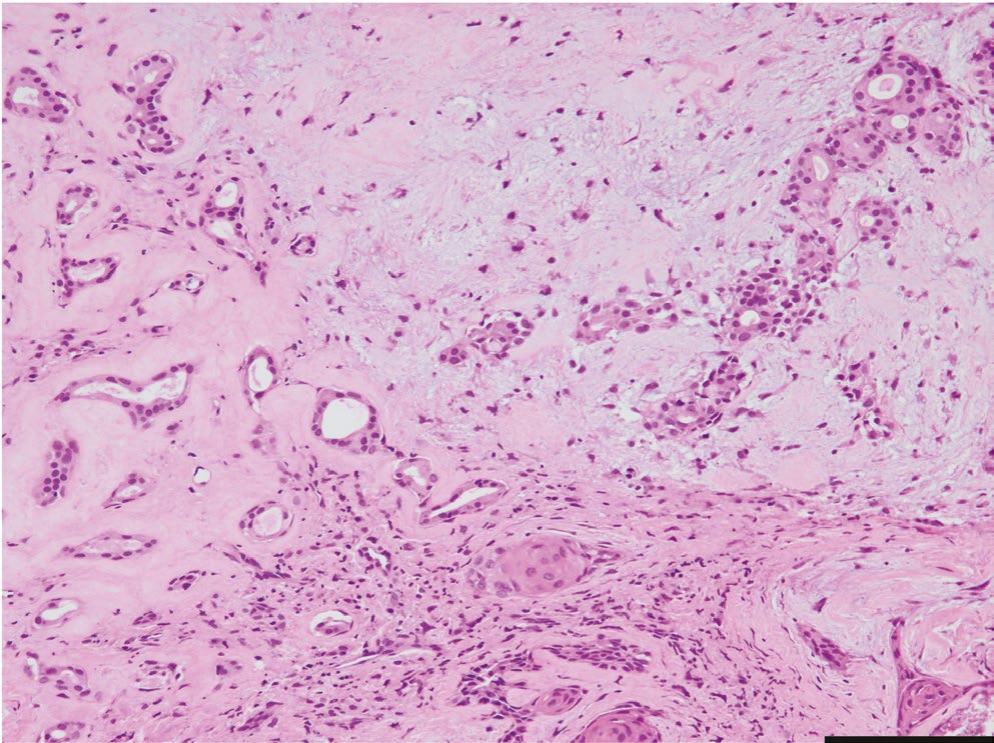
Histology of FNAB specimen section stained with HE. The tumor was composed of epithelial, myoepithelial, and stromal components, lacking notable cellular atypia. Bar, 200 μm. FNAB, fine‐needle aspiration biopsy; HE, hematoxylin and eosin

**Figure 4:**
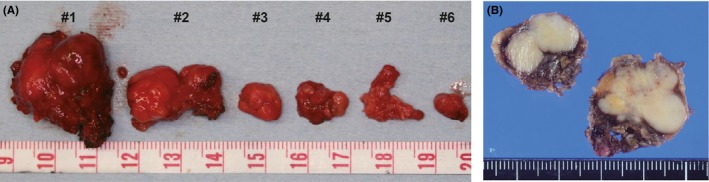
Macroscopic findings of the surgically resected nodules. A, The resected tumor lesion consisted of six various‐sized nodules. B, The cut surface of two (#1 and #2) nodules after formalin fixation. The tumor was solid and grayish‐white in color

A 60‐year‐old Japanese woman harbored an asymptomatic mass in the left buccal mucosa. She had surgical history in the same portion approximately 30 years ago, but the details were unknown. She noticed the mass in the buccal 5 years ago. She was referred to the Oral and Maxillofacial Surgery Department at Miyazaki University Hospital, because the mass had slowly increased in size. Her face was asymmetrical because of the swelling of the left cheek. In the findings of the oral cavity, the buccal mucosa of the left side was elastic hard and normal in color. Ulcerative changes were not found, and there was no sign of inflammation. Laboratory data were within the normal ranges. Nonenhanced computed tomography (CT) showed various‐sized nodules at the left cheek (Figure 1). These nodules were located in front of the masseter muscle and the mandibular ramus. Neither calcification nor adipose tissue was observed in the nodules. These nodules were separated from the parotid duct. No swelling of the lymph nodes was detected. Magnetic resonance imaging (MRI) revealed heterogeneous nodules with low intensity on T1‐weighted imaging (Figure 2A). The signal intensity of nodules was similar to that of a muscle tissue. On T2‐weighted imaging, multiple nodules showed mixed low and high signals, and the high‐intensity signal portions suggested the existence of multiple cyst‐like changes (Figure 2B). CT and MRI findings of this lesion suggested PA, and FNAB was performed from the left buccal mucosa. The biopsy specimen revealed mixed proliferation of epithelioid and myoepithelioid tumor cells lacking notable cellular atypia. These tumor cells proliferated showing a tubular or sheet‐forming fashion accompanying myxoid stroma in places (Figure 3). The histopathological diagnosis based on the FNAB specimen was benign PA. The patient then underwent tumor resection surgery using an intraoral approach under general anesthesia. The excised specimens were six, various‐sized, solid tumor nodules (Figure 4A). The cut surfaces of the nodules were grayish‐white, and necrosis was partly observed (Figure 4B).

**Figure 5:**
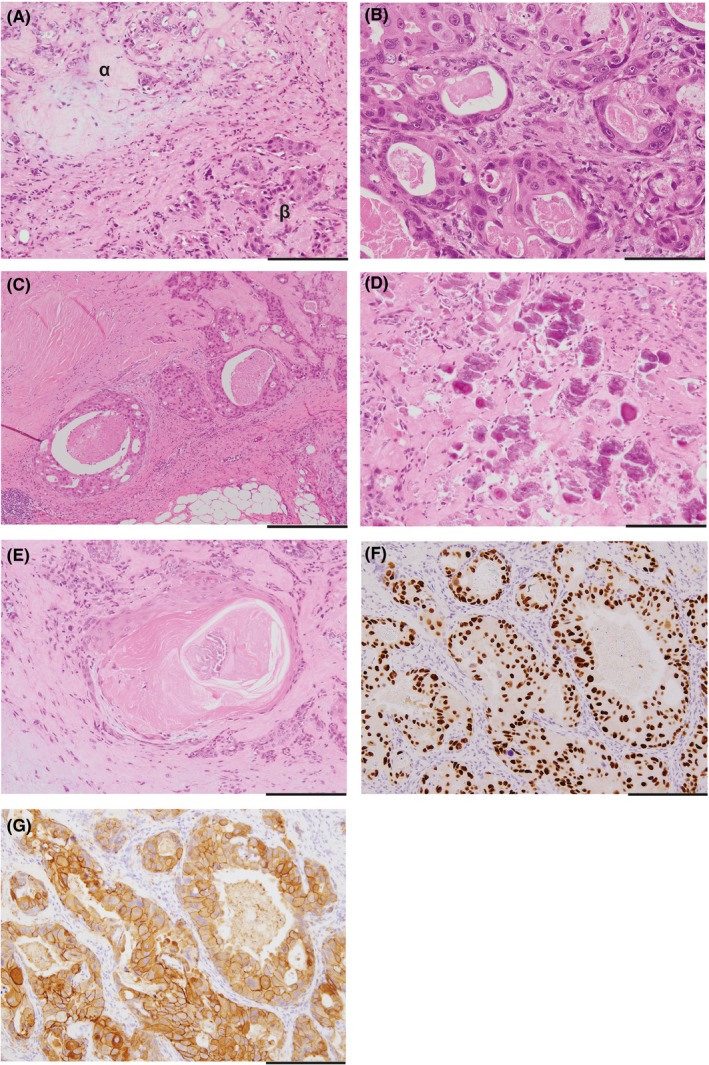
Histology of the resected tumor in #1. A, The tumor was composed of pleomorphic adenoma (α) and malignant components (β) (HE). Bar, 500 μm. B, High magnification image of malignant component (HE, ×200). Bar, 100 μm. C, Roman bridge‐like cribriform pattern with comedo‐necrosis (HE, ×40). Bar, 400 μm. D, Squamous metaplasia (HE, ×100). Bar, 200 μm. E, Calcified materials (HE, ×100). Bar, 200 μm. F, G, Immunohistochemical staining for AR and HER2. The tumor cells were strongly positive for AR (F) and HER2 (G). Bars, 200 μm. HE, hematoxylin and eosin; AR, androgen receptor; HER2; human epidermal growth factor receptor 2

Histologically, the tumor was composed of a mixture of epithelioid or myoepithelioid tumor cells and intermingled myxoid or hyalinized stromal component, which were similar to those observed in the FNAB specimen. However, in two of the six nodules, atypical epithelial cells with large hyperchromatic nuclei and prominent nucleoli were proliferated to form tubules (Figure 5A and 5). Partly, Roman bridge‐like cribriform features were observed accompanying comedo‐necrosis (Figure 5C). Calcified bodies and squamous metaplasia were intermingled (Figure 5D and 5). The cancer lesions were surrounded by the PA tissue or a capsule‐like fibrous tissue. The atypical cells tested positive for cytokeratin5/6 and p53 (focal) but tested negative for glial fibrillary acidic protein, alpha‐smooth muscle actin, and S100. They tested strongly positive for the androgen receptor and human epidermal growth factor receptor 2 (Figure 5F and G). The Ki‐67 labeling index was 19.8% in the hot spots. We considered that these atypical cells represented malignant transformation and the final pathologic diagnosis was Ca‐ex‐PA arising from the buccal mucosa with a salivary duct carcinoma (SDC) component. On postoperative MRI, the tumor remained. Therefore, she underwent tumor reresection, selective neck dissection, and forearm flap surgery under general anesthesia. Two years and 5 months postoperatively, the patient was disease‐free.

## 3 DISCUSSION

Ca‐ex‐PA is defined as a PA from which an epithelial malignancy is derived.^3^ Approximately 1.6%‐7.5% of PA contains a malignant component in its natural course.^6^ The period from onset to diagnosis ranges from several months to over 20 years^7^, ^8^: The longer the period from the occurrence of PA, the higher the probability of malignant change. Indeed, the probability of malignant change has been reported as 1.6% in cases of <5 years and 9.6% in cases of more than 15 years.^9^ In our case, the patient noticed swelling of her oral mucosa 5 years before the surgery. Moreover, this site had a history of prior surgery around 30 years ago, although the exact details were not known. Because this case involved a multinodular lesion, it is possible that the previous lesion was associated with PA, and the recurrence occurred as Ca‐ex‐PA after more than 35 years. The commonest site of Ca‐ex‐PA is the parotid gland, followed by the submandibular gland. Regarding the minor salivary glands, the hard and soft palates are usual sites for this tumor. Ca‐ex‐PA arising from the buccal mucosa is extremely rare, and thus far, only seven cases have been reported in the English literature.^10^, ^11^, ^12^

FNAB and FNAC are widely used for the preoperative diagnosis of breast and prostate tumors, and they are frequently used for salivary gland tumors including Ca‐ex‐PA. Compared to excisional biopsy, FNAB is quick, simple, and minimally invasive. Moreover, FNA studies significantly reduce complications, including facial nerve damage, tumor implantation, fistula formation, and tissue reactions, which make subsequent surgery difficult.^13^ Meanwhile, a serious matter remains in FNA studies. Several studies have demonstrated that the sensitivity of FNAC when diagnosing Ca‐ex‐PA is 29%‐50% and that the reliability has never been shown to be high.^14^, ^15^, ^16^ In salivary gland tumors, the sensitivity and specificity of FNAB were 68.2% and 87.7%, respectively.^17^ In our case, the FNAB specimen contained only a PA component, and the carcinoma component was detected only after surgery because of a sampling error. Our case involved a multinodular lesion, in which the malignant component was present only in two of six nodules.

In the malignant component of Ca‐ex‐PA, adenocarcinoma not otherwise specified is the commonest histological subtype, followed by SDC and myoepithelial carcinoma.^8^, ^18^ Rare cases such as those of adenoid cystic carcinoma, mucoepidermoid carcinoma, adenosquamous cell carcinoma, epithelial‐myoepithelial carcinoma, and sarcomatoid carcinoma can be identified.^18^ In this case, the carcinoma component was SDC. SDC shows aggressive clinical behavior and poor clinical outcomes.^19^ Recently, prognostic factors of SDC have been extensively studied, and tumor size, positive margins, perineural invasion, local recurrence, lymph node metastasis, and distant metastasis were reported as poor prognostic factors.^20^ A prognostic survey of SDC has been performed mainly for those arising in the major salivary glands, particularly the parotid gland. Meanwhile, the investigation for SDC prognosis in the minor salivary glands may be insufficient.^21^ HER2 has been reported to be positive in approximately 40% of SDC,^22^, ^23^ and the overexpression of HER2 and tumor protein p53 is likely associated with the poor clinical course of SDC.^20^

Regardless of the histological subtype of the malignant component, evaluation of invasion is one of the most important prognostic criteria. Ca‐ex‐PAs can be subclassified into three types: noninvasive (intracapsular carcinoma), minimally invasive (≤1.5 mm extracapsular invasion), and widely invasive (>1.5 mm extracapsular invasion).^1^ This threshold has been adopted by the World Health Organization Classification of Salivary Gland Tumours,^24^ but this threshold is still a matter of debate.^24^, ^25^, ^26^ Verification based on appropriate evidence is necessary for the future.

## CONFLICT OF INTEREST

None declared.

## AUTHOR CONTRIBUTIONS

KY: drafted the manuscript. YK and YY: contributed to patient management. HT, YA, and YS: conducted histopathological investigations. HK: contributed to writing the final manuscript.
